# Diet-Related Health Recommender Systems for Patients With Chronic Health Conditions: Scoping Review

**DOI:** 10.2196/77726

**Published:** 2026-01-14

**Authors:** Xiaolan Dong, Bei Yun, Anni Pakarinen, Zhuting Zheng, Hao Niu, Tian Jin, Changrong Yuan, Jingting Wang

**Affiliations:** 1 Department of Nursing Science, University of Turku, Turku, Finland; 2 School of Nursing, Fudan University, Shanghai, China; 3 Faculty of Nursing and Physiotherapy, University of Lleida, Lleida, Spain; 4 School of Nursing, Naval Medical University, 800 Xiangyin Road, Shanghai, 200043, China, 86 02181871492

**Keywords:** Health Recommender System, Diet-related Health Recommender System, chronic health conditions, diet, scoping review, PRISMA, HRS

## Abstract

**Background:**

Diet-related Health Recommender Systems (HRSs) have gained attention for their potential to provide personalized dietary guidance, particularly for patients with chronic conditions. However, studies on diet-related HRSs in health care are relatively limited.

**Objective:**

This scoping review aims to present the state of current research on diet-related HRSs for patients with chronic health conditions, identify existing gaps, and suggest future research directions.

**Methods:**

The scoping review was conducted following the Arksey and O’Malley framework and was reported in accordance with the PRISMA-ScR (Preferred Reporting Items for Systematic Reviews and Meta-Analyses Extension for Scoping Reviews) guidelines. The literature search was conducted in October 2024 across 6 English databases (PubMed, Medline, Embase, Web of Science Core Collection, IEEE Xplore, and CINAHL) and 4 Chinese databases (SinoMed, CNKI, Wanfang, and VIP). Studies focusing on diet-related HRSs for patients with chronic conditions were included.

**Results:**

Fifteen studies published between 2010 and 2024 from 9 countries were included. Diet-related HRSs mainly target adults with chronic diseases, with 9 systems (60%) including users with diabetes and 6 (40%) including users with hypertension. Nine studies (60%) described functional structures, which were categorized into 4 components: user information, food or diet recommendations, knowledge and decision support, and data management with additional functions. Recommended content was categorized into 5 types: food (n=6, 40%), recipes (n=4, 26.67%), diet plans or meal plans (n=3, 20%), recipes and food (n=1, 6.67%), and meals (n=1, 6.67%). Recommendation methods included constraint-based (n=6, 40%), focusing on patients’ dietary restrictions; preference-based (n=5, 33.33%), considering patients’ food preferences; and hybrid (n=4, 26.67%), combining both approaches. Of all recommendation technologies, most studies (n=13, 86.67%) applied hybrid approaches, enabling more robust personalization. For the data used for training, 13 studies (86.67%) explicitly mentioned the data sources, and 10 studies’ (66.67%) data came from professional organizations and websites. The recommendation process followed a structured workflow. Twelve studies (80%) evaluated diet-related HRSs using either online or offline methods, while accuracy (n=9, 60%) has been the most common evaluation criterion. However, no studies went deeper into how these systems affected users’ dietary behaviors over time.

**Conclusions:**

Diet-related HRSs have the potential to deliver personalized dietary support for patients with chronic diseases, but current systems show key gaps. Future development must adopt user-centered design, provide practical and actionable dietary guidance, and use hybrid recommendation techniques to increase precision and clinical relevance. Standardized evaluation methods and real-world, long-term studies are essential to evaluate the impact of diet-related HRSs on dietary behavior and health outcomes. Addressing these needs will enable diet-related HRSs to become reliable tools for chronic disease management and patient-centered care.

## Introduction

Dietary management is crucial to overall health, especially in the context of global trends where poor dietary habits have become a significant factor contributing to weight-related issues [1]. Statistics from the latest Global Nutrition Report indicated alarming rates of overweight and obesity among adults, with 40.8% of adult (18 years or older) women and 40.4% of adult men affected. Conversely, 9.1% of adult women and 8.1 % of adult men were underweight [2]. Improper dietary management not only affects body weight but also contributes to the development of diabetes [3,4], hypertension [5], cardiovascular diseases [6], chronic kidney disease [7], and inflammatory bowel disease [8], among others.

The Scientific Research Report on Dietary Guidelines for Chinese Residents (2021) highlighted that the overweight and obesity rates among children younger than 6 years old and those aged 6‐17 years were 10.4% and 19.0%, respectively [9]. Among residents aged 18 years and older, the overweight rate was 34.3% and the obesity rate was 16.4%, with 50.7% of adults being overweight or obese [9]. Overweight and obesity are significant risk factors for cardiovascular diseases, diabetes, cancer, and other chronic health conditions [9].

Poor dietary habits contribute to both the onset and progression of these chronic conditions. Therefore, scientific and effective diet management is essential for ensuring proper nutrient intake, which directly enhances individual immunity, halts disease progression, impacts health status, and supports recovery from chronic health conditions, thereby improving overall health outcomes [7,10,11]. Moreover, good diet management regulates sleep and mood, reduces fatigue, and comprehensively enhances overall health [12,13]. In February 2024, China’s National Health Commission released the “Dietary Guidelines for Adults with Hyperuricemia and Gout (2024 Edition),” “Dietary Guidelines for Adult Obesity (2024 Edition),” “Dietary Guidelines for Childhood and Adolescent Obesity (2024 Edition),” and “Dietary Guidelines for Adults with Chronic Kidney Disease (2024 Edition)” [14]. These guidelines aim to prevent and control the occurrence and progression of chronic diseases among the Chinese population through dietary management.

Patients with chronic health conditions often need to adjust their diets based on their specific health status to manage health conditions effectively and enhance the overall quality of life. When patients are required to follow dietary restrictions due to their chronic health condition, it is important for the patient to distinguish whether certain foods are permissible, ensure that the cooking methods meet the essential requirements, evaluate whether portion sizes are appropriate, and strive to maintain a balanced diet to reduce the risk of malnutrition and other issues caused by dietary limitations [15]. Therefore, these patients require targeted and personalized guidance to help them implement scientific and feasible dietary management practices.

Recommender Systems (RSs) are software tools that provide suggestions or recommendations of items to users, such as products, services, information, or content, based on their preferences, interests, and past behaviors [16], which have been used in several domains such as e-commerce, e-learning, e-tourism, or eHealth. To generate recommendations, RSs commonly rely on a set of foundational recommendation technologies. Collaborative filtering (CF) is one of the most widely applied recommendation technologies, which provides recommendations for users by using the known preferences of other users with similar behaviors [17]. In contrast, content-based (CB) methods recommend items similar to those a given user has previously liked [18]. Unlike CF and CB, which rely on large historical rating datasets, knowledge-based (KB) approaches produce suggestions by leveraging domain knowledge, expert rules, and explicit user constraints [19]. Knowledge graph (KG)-driven recommendation further enhances personalization by representing domain knowledge in graph structures to generate precise suggestions [20]. Finally, hybrid recommendation (HyR) integrates 2 or more strategies to maximize their strengths and mitigate individual limitations [21].

Health Recommender Systems (HRSs) are also one of the RS’s important application scenarios. HRSs offer the potential to motivate and engage users to change their behavior by sharing better choices and practical knowledge based on observed user behavior [22]. HRSs have been applied in health care in recent years, in areas such as mental health [23], hearing aid usage [24], health education [25], physical activity [26], and diet-related health [27].

Diet-related HRSs are software tools that use personalized data to provide tailored food recommendations from a wide range of options [28]. Diet-related HRSs present promising solutions to the issues of information overload and limited food choices, which contribute significantly to diet-related health problems [29]. By using personalized data, diet-related HRSs offer tailored food recommendations that take into account users’ taste preferences, dietary needs, and medical conditions [30]. Such systems can filter and sort food options [27], fostering a better understanding of dietary health and increasing user engagement [31]. Additionally, diet-related HRSs can enhance dietary outcomes by providing nutrition assessments and offering healthier meal plans and recipes [28].

While diet-related HRSs have been explored within the field of information and communication technology, research on their application in chronic disease dietary management is still limited, and there is a lack of comprehensive literature reviews in this area. To address these gaps, this study adopted a scoping review methodology to review the research status quo of diet-related HRSs for individuals with chronic conditions. In this review, diet-related HRSs are defined as systems specifically designed for patients with chronic health conditions that require disease-related dietary restrictions. The target populations, function structures, recommendation content, implementation of recommendation features, and evaluation of the diet-related HRSs were analyzed to provide a reference for health care researchers seeking to design more effective and user-friendly systems.

## Methods

### Overview

Scoping reviews assess the extent of the research, range, and nature, identify gaps, determine systematic review value, and disseminate research findings [32]. A scoping review methodology is used to systematically map available research on the broad, complex, and emerging research question [32]. In emerging research fields, a lack of randomized controlled trials may impede formal systematic reviews or meta-analyses. Scoping studies can address diverse questions and incorporate a range of study designs, making them ideal to complement clinical trial findings. The scoping review was conducted following the Arksey and O’Malley framework [32] and was reported in accordance with the PRISMA-ScR (Preferred Reporting Items for Systematic Reviews and Meta-Analyses Extension for Scoping Reviews) guidelines [33] and was structured according to the following outlined steps.

### Stage 1: Identification of the Research Question

The research question was identified after an initial review of the literature and a discussion within our research team. The key research questions that guided the review were as follows:

1. What is the current state of research on diet-related HRSs for patients with chronic health conditions?
2. What are the target users for diet-related HRSs for patients with chronic health conditions?
3. What are the function structures in diet-related HRSs for patients with chronic health conditions?
4. What types of recommendation content are provided by diet-related HRSs for patients with chronic health conditions?
5. How are recommendation features implemented in diet-related HRSs for patients with chronic health conditions?
6. How are diet-related HRSs for patients with chronic health conditions evaluated?

### Stage 2: Identifying Relevant Studies

Before searching, the research team worked collaboratively in making decisions about inclusion and exclusion criteria and in planning the initial search strategies to comprehensively identify the relevant literature. The final search strategies were developed with the assistance of a health science librarian (Multimedia Appendix 1). The literature search was performed by 3 researchers in October 2024, covering 6 English databases (PubMed, Medline, Embase, Web of Science Core Collection, IEEE Xplore, and CINAHL) and 4 Chinese databases (SinoMed, CNKI, Wanfang, and VIPC). Relevant studies were searched from January 2010 to October 2024 to answer the above research questions (1-6). An example of the search strategy performed in Web of Science Core Collection is presented here: (recommender system* OR hybrid recommendation* OR collaborative filtering OR content based recommendation* OR recommendation* system* OR knowledge based recommendation*) AND (recipe* OR diet* OR food OR eat* OR nutrition*). As the search proceeded, additional terms were suggested by experts to potentially modify the question, but new research did not result in additional data, and the question did not change. Duplicate references were filtered out using EndNote. In addition to database search, hand searching was conducted by screening the reference lists of all included articles and relevant review papers to identify additional eligible studies. We also manually checked key journals in the field and conference proceedings to ensure comprehensive coverage.

### Stage 3: Study Selection

All studies searched were independently assessed by 2 authors (XD and BY) based on the inclusion criteria listed below, and discrepancies were verified by a third author (JW). Studies were eligible for inclusion in this review if all the following criteria were met: (1) the recommended information was related to at least one of the following: food, meal plan, diet plan, and recipe; (2) the study applied personalized recommendation strategy; (3) the recommendations were generated using algorithmic and technological methods; (4) the study population comprised patients with chronic health conditions; and (5) the study was published in a peer-reviewed journal or conference proceeding. The exclusion criteria included: (1) the recommendation unrelated to human health (eg, study focusing on animal health [34]), (2) studies reporting the same RSs were considered duplicates; only the most recent or most comprehensive publication was retained (eg, the latest published study [35] was included, while the earlier one [36] was excluded), and (3) full-text articles not in English or Chinese language.

### Stage 4: Charting the Data

Decisions regarding the information to be recorded from the primary studies were made through discussions within the research group. Subsequently, a structured chart was developed to collate, summarize, and share the extracted data. A descriptive-analytical narrative approach was used to extract and chart the data from the selected articles [32,37]. Three researchers (XD, BY, and ZZ) independently extracted the data and performed coding, with the other two authors (HN and JW) verifying accuracy. All discrepancies were resolved by consensus. The following details were documented for each included study to answer the research questions: (1) nationality and publication year (Multimedia Appendix 2); (2) target users; (3) function structures; (4) recommendation content; (5) recommendation method, recommendation technology, data of training set, and recommendation process; and (6) evaluation method, evaluation criteria, test set, or evaluation sample size.

### Stage 5: Collating, Summarizing, and Reporting the Results

The scoping review methodology aimed to summarize the breadth and depth of the existing literature. At this stage, an overview of the characteristics of all included articles was collated, summarized, and reported. Initially, a basic numerical summary of the studies, including the extent, nature, and distribution of the articles, was presented. As this was a scoping review, the critical appraisal of the quality of the included studies was not conducted. However, efforts were made to map the diversity and variety of diet-related HRSs based on factors such as the characteristics of target users, function structure, recommendation content, and other factors. This process facilitated researchers in reaching conclusions about the key characteristics of research in this field and provided insights for future studies.

## Results

### Overview

**Figure 1.**
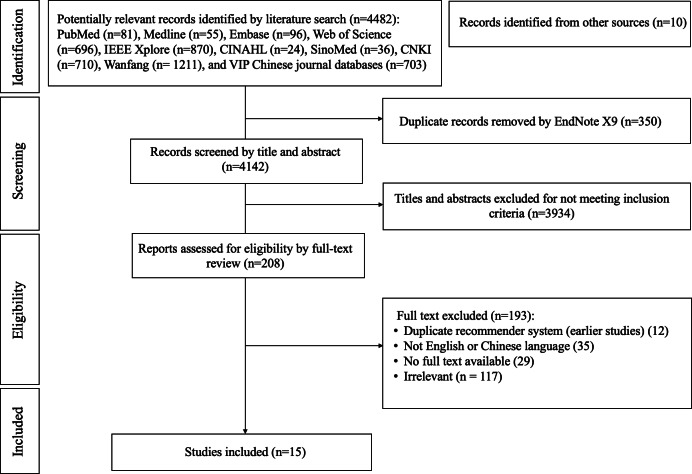
Flow diagram according to the PRISMA (Preferred Reporting Items for Systematic Reviews and Meta-Analyses) guidelines.

In total, 4492 published studies were identified in the searching process (Figure 1). EndNote X9 was used to exclude 350 duplicates, and 4142 studies were excluded based on a review of their titles and abstracts. The remaining 208 studies were searched for full text. Ultimately, 193 were excluded based on the exclusion criteria, and 15 studies [35,38-51] were included and analyzed.

### The Current State of Research on Diet-Related HRSs for Patients with Chronic Health Conditions

The publication years and the countries of the included studies are shown in Multimedia Appendix 2. The studies were published between 2010 and 2024, and there has not been a noticeable increase in their publication over the past 5 years. The articles originated from 9 countries (based on the first author’s affiliations), with the top 3 being China (n=5, 33.33%) [39,40,43,44,49], India (n=2, 13.33%) [41,47], and Pakistan (n=2, 13.33%) [38,48].

### Target Users for Diet-Related HRSs for Patients with Chronic Health Conditions

Among the 15 included studies [35,38-51], only 2 reported the age of the target users, which were 18‐80 years [50] and older than 65 years [39], respectively. The target users and patients’ chronic health conditions are shown in Table 1. Diet-related HRSs primarily target adults with chronic diseases. Among chronic diseases, diabetes and hypertension were the most commonly targeted by diet-related HRSs, with 9 systems (60%) including users with diabetes and 6 (40%) including users with hypertension.

**Table 1.** The characteristics of target users for diet-related Health Recommender Systems.

Variable	Studies, n (%)	References
Target users		
Patients with a single disease	6 (40)	[38,40,41,44,46,51]
Patients with complex diseases	4 (26.67)	[39,43,47,48]
Both healthy people and patients	5 (33.33)	[35,42,45,49,50]
Patients’ chronic health conditions		
Diabetes	9 (60)	[35,38,42,45-49,51]
Hypertension	6 (40)	[35,40,45,47-49]
Cancer	3 (20)	[41,42,44]
Obesity	3 (20)	[42,47,50]
Kidney disease	3 (20)	[35,47,48]
High cholesterol	2 (13.33)	[47,49]
Metabolic syndrome	1 (6.67)	[43]
Osteoporosis	1 (6.67)	[42]
Iron deficiency	1 (6.67)	[48]
Cardiovascular diseases	1 (6.67)	[42]
Chronic dental problems	1 (6.67)	[42]
Unspecified geriatric diseases	1 (6.67)	[39]

### The Function Structures in Diet-Related HRSs for Patients with Chronic Health Conditions

Among the 15 studies included [35,38-51], 9 (60%) [35,38-45] studies described function structures. The function structures described in these studies varied, but certain recurring components were consistently noted, which could be summarized into 4 major components: user information, food or diet recommendations, knowledge and decision support, and data management and additional functions. Detailed information is shown in Table 2.

**Table 2.** The function structures in diet-related Health Recommender Systems.

Variable	References
User information	
Basic user information	
Personal/user profile	[35,38,39]
User information	[40]
History	[35,41,42]
Personal health information	
Physical activity	[38]
Health management report	[43]
Diseases	[44]
Symptoms	[44]
Diagnoses	[41]
Food or diet recommendations	
Food filter and security	[35]
Food recommendations or suggestions	[38,45]
Weekly meal plans	[42]
Diet guides	[39,40]
Knowledge and decision support	
Chronic kidney disease calculator	[35]
Food	[38]
Nutrient search engines	[45]
News and related sites	[45]
Ingredients	[44]
Nutritional expert knowledge	[39]
Clinical practice and nutrition guidelines	[39]
Knowledge base	[39]
Diet charts	[41]
User ratings	[42]
Data management and additional functions	
Data entry	[38]
Data management	[40]
Reminders	[35]
Real-time interaction	[43]
Settings	[40]
Changing details	[41]

### The Types of Recommendation Content Provided by Diet-Related HRSs for Patients with Chronic Health Conditions

The recommended content was divided into 5 categories. Detailed information is shown in Table 3.

**Table 3.** The types of recommendation content provided by the diet-related Health Recommender Systems.

Recommended content	Studies, n (%)	References
Food	6 (40)	[35,41,46-49]
Recipe	4 (26.67)	[38,42,44,45]
Diet plan or meal plan	3 (20)	[40,43,50]
Both recipe and food	1 (6.67)	[51]
Meal	1 (6.67)	[39]

### The Implementation of Recommendation Features in Diet-Related HRSs for Patients with Chronic Health Conditions

Table 4 presents detailed information on the implementation of recommendation features in the reviewed diet-related HRSs. Recommendation methods were inductively classified into 3 categories: constraint-based, preference-based, and hybrid. In this review, constraint-based refers to recommendations based on the patient’s condition-related dietary restrictions, while preference-based refers to recommendations based on the patient’s dietary preferences. Hybrid refers to methods that incorporate both constraint-based and preference-based approaches. Among the 15 included studies [35,38-51], 6 (40%) used constraint-based methods [41,43,46,47,49,51], 5 (33.33%) applied preference-based methods [39,40,44,45,50], and 4 (26.67%) adopted hybrid methods [35,38,42,48].

**Table 4.** The implementation of recommendation features in diet-related Health Recommender Systems.

Author [reference], publication year	Recommendation method	Recommendation technology	Data of the training set	Recommendation process
Phanich et al [46], 2010	Constraint-based	Hybrid recommendation	Data were selected from the nutrition division, Ministry of Public Health, to form the training dataset (n=290^a^)	The dataset was grouped by the system and categorized according to food characteristics and nutrition for diabetes (normal food, limited food, and avoidable food). Features were extracted by nutrient ranking. Food clustering was analyzed. Relevant food items were ranked based on the minimum distance, extracted, and recommended in ascending order from the ranking.
Arwan et al [51], 2013	Constraint-based	Hybrid recommendation	Data were selected from some references of foods and enriched with the information from nutrition experts to form the training dataset (n=NR^b^).	The food ontology and calorie food ontology were developed. The rule for classifying or grouping data categories within the ontology was developed. The food menu search feature was built. An experiment was conducted to test whether the system could recommend correctly.
Faiz et al [38], 2014	Preference-based and constraint-based	Hybrid recommendation	Data were selected from standard and well-known resources such as the US Department of Agriculture and MyFitnessPal to form the training dataset (n=NR^b^).	The domain ontologies (personal health profile, food, and diseases) were built. The ontologies were integrated and rule terms were defined. The diet was recommended to the user at the predefined time.
Ting et al [45], 2014	Preference-based	Hybrid recommendation	Data were selected from the Food Composition Database and Japan Preventive Association of Lifestyle-related Disease to form the training dataset (n=NR^b^).	A query with the user data was sent to the system. Recipes were obtained from the database through a search. Recipes that did not suit the current health status or match the user’s preferences were filtered out. Five recipes were recommended from the filtered set.
Chen et al [49], 2015	Constraint-based	Hybrid recommendation	Data were collected from the Taiwan Area Food Nutrition Database website published by the US Food and Drug Administration to form the training dataset (n=NR^b^).	The system collected personal information and classified the user’s nutrients. The patient’s dietary records were imported into the nutrition expert knowledge system. The personal information was imported into the personal disease ontology. The nutrient data were analyzed by the expert knowledge system. Suitable foods for the user were inferred.
Tseng et al [43], 2015	Constraint-based	Hybrid recommendation	—^c^	The patient’s vital signs were collected and transmitted. The risk was evaluated and reported. A diet plan was generated and recommended to the patient.
Elsweiler et al [50], 2015	Preference-based	Hybrid recommendation	Data were selected from a self-created food portal website, including users (n=148^d^) and recipes (n=957^a^) to form the training dataset.	The user’s nutritional requirements were established. Recipe ratings were estimated based on each profile. Recipes were combined. Recipes were established if the combination met the requirements.
Rehman et al [48], 2017	Preference-based and constraint-based	Hybrid recommendation	Data were selected from the official website of the Composition of Foods Integrated Dataset (CoFID) to form the training dataset (n=3400^a^).	A cloud-based food recommender system, named Diet-Right, was developed based on users’ pathological reports. The ant colony algorithm was used to generate an optimal food list. Suitable foods were recommended according to the values of pathological reports.
Agapito et al [35], 2018	Preference-based and constraint-based	Hybrid recommendation	—^c^	The user profile was created by giving specific questions about clinical parameters. All changes made by the user were saved, allowing the data to be used for monitoring the user’s health status. After the user was profiled, typical foods that could be consumed by the user were recommended.
Rathi et al [41], 2019	Constraint-based	Hybrid recommendation	Data were selected from the UC Irvine Machine Learning Repository (including the Liver Patient Dataset, Heart Disease Dataset, Diabetes Dataset, Breast Cancer Dataset, and Thyroid Dataset).	The dataset was collected and preprocessed from different sources. The system was built to detect diseases and recommend diets accordingly.
Manoharan et al [47], 2020	Constraint-based	Hybrid recommendation	Data were collected from 50 patients through the internet and hospitals to form the training dataset (n=50^d^).	Data were collected from the internet and hospitals. Features were sorted, preprocessed, and encoded, and the food data were segregated based on similarities. Food was recommended. The system was trained, tested, and cross-validated.
Qi et al [40], 2021	Preference-based	Hybrid recommendation	Data were selected from hospital meal history records and split into training and testing sets in a 7:3 ratio (n=718^a^).	A MySQL database was used to implement data dictionary management. The rule extraction module was implemented with the knowledge base management system. A meal plan was generated and recommended.
Tang et al [44], 2023	Preference-based	Knowledge graph	Data were crawled from recipe websites and manually extracted from textbooks to form the training dataset (n=NR^b^).	The embedded representation of items was enhanced through message-passing and update functions on node features. The influence of time on users’ taste preferences was considered. Long Short-Term Memory (LSTM) networks were introduced to dynamically adjust users’ personal taste preferences.
Zioutos et al [42], 2024	Preference-based and constraint-based	Hybrid recommendation	Data were selected from a large real-world dataset of recipes to form the training dataset (n=2,774,676^a^).	The user’s health history was analyzed. Similar users were identified. Personalized recipe recommendations were provided. Dynamically adaptable adjustments were made.
Xu et al [39], 2024	Preference-based	Knowledge graph	Data were selected from a community survey (n=96^d^) and a website of Chinese cuisine recipes and eating history (n=180^a^) to form the training dataset.	The FoodKG was constructed. User profiles related to older adults’ dietary behaviors were built. Personalized meal recommendation algorithms were developed, including candidate dish generation and combo meal recommendations.

a The sample size of subjects including a recipe, food, and diet plan or meal plan.

b NR: not reported.

c Not available.

d The sample size of the population.

While the recommendation technologies were coded according to terms reported in the studies. The recommendation technology included: KG (n=2, 13.33%) [39,44] and HyR (n=13, 86.67%) [35,38,40-43,45-51].

For the data of the training set, 13 studies (86.67%) [38-42,44-51] explicitly mentioned the data sources of the training set, while the other 2 studies (13.33%) [35,43] did not specify this information. The data sources used in the reviewed studies varied widely and can be categorized into 4 main types: authoritative government and institutional databases (n=5, 33.33%) [38,45,46,48,49], academic databases and publicly available datasets (n=2, 13.33%) [39,41], expert and hospital data (n=3, 20%) [40,47,51], and recipe and user-generated data (n=3, 20%) [42,44,50].

The recommendation process followed a structured workflow, integrating advanced technologies to generate more accurate, adaptive, and context-aware dietary recommendations. The structured workflow included user profiling, integration of structured knowledge (such as food databases or ontologies), personalized filtering and matching based on health conditions and preferences, and ranking of suitable options for final recommendation. While this core process was consistent, 6 systems incorporated advanced technologies to enable more accurate, adaptable, and context-aware dietary recommendations, such as ontology-based reasoning [51], optimization algorithms (eg, ant colony [48]), dynamic modeling using Long Short-Term Memory networks [44], expert rule systems [39,49], and the use of KGs such as FoodKG [39].

### The Evaluation of Diet-Related HRSs for Patients with Chronic Health Conditions

Among the 15 included studies [35,38-51], 12 studies (80%) [35,39-44,46-49,51] evaluated the diet-related HRSs, while the remaining 3 studies (20%) [38,45,50] did not conduct an evaluation. Table 5 presents detailed information on the evaluation criteria, evaluation method, and test set or evaluation sample size of the 12 studies [35,39-44,46-49,51].

**Table 5.** The evaluation of diet-related Health Recommender Systems (n=12).

Author [reference], publication year	Evaluation criteria	Evaluation method	Test set or evaluation sample size
Phanich et al [46], 2010	Acceptance, usability, and accuracy	Online	Nutritionists (n=NR^a^)
Arwan et al [51], 2013	Accuracy	Offline	Ontology patient instances (n=30^b^)
Chen et al [49], 2015	Accuracy	Online	One older adult from a silver-haired home and dietitians (n=NR^a^)
Tseng et al [43], 2015	Feasibility	Online	Patients (n=NR^a^)
Rehman et al [48], 2017	Accuracy	Offline	Same as the training set
Agapito et al [35], 2018	Accuracy and usability	Online	Patients with CKD^c^ (n=20^b^); healthy people (n=20^b^)
Rathi et al [41], 2019	Accuracy	Offline	Same as the training set. The dataset was split into training:testing 75:25 (n=9326^d^)
Manoharan et al [47], 2020	Accuracy	Offline	Same as the training set
Qi et al [40], 2021	Efficiency and satisfaction	Online	Patients (n=37^b^) and nutritionists (n=3^b^)
Tang et al [44], 2023	Accuracy	Offline	Same as the training set
Zioutos et al [42], 2024	Accuracy and usability	Online	40 existing users of the food.com website: patients (n=20^b^) and healthy people (n=20^b^)
Xu et al [39], 2024	Effectiveness	Online	Community-dwelling older adults (n=96^b^): tracked group (n=34^b^) and an untracked group (n=62^b^); A total of 91 participants (94.79%) were diagnosed with chronic conditions

a NR: not reported.

b The sample size of the population.

c CKD: chronic kidney disease.

d The sample size of subjects including a recipe, food, and diet plan or meal plan.

The evaluation criteria were extracted directly from the included studies, including: accuracy (n=9) [35,41,42,44,46-49,51] refers to the extent to which the system’s predictions of patients’ preferences or nutritional needs match their actual dietary preferences or intake; usability (n=3) [35,42,46] refers to the ease with which patients can, with little or no assistance, successfully operate the system to receive recommendations, navigate, and interact with its interface; acceptance (n=1) [46] refers to the degree to which patients are willing to receive and use the dietary recommendations provided by the system; satisfaction (n=1) [40] refers to patients’ subjective sense of contentment or evaluation of the overall usefulness and experience of the recommendations, measured through participants’ comprehensive comparison of system-generated meal plans with manually designed ones; efficiency (n=1) [40] refers to the amount of time, steps, or cognitive effort required for patients to obtain appropriate dietary recommendations from the system; feasibility (n=1) [43] refers to the practicality of deploying the recommendation system in real-life settings; and effectiveness (n=1) [39] refers to the actual positive impact of the system in real-life contexts on patients’ dietary behavior changes or health outcomes. Four studies [35,40,42,46] included more than one evaluation criterion.

The evaluation methods included: online evaluation (n=7) [35,39,40,42,43,46,49] and offline evaluation (n=5) [41,44,47,48,51]. Among the 7 studies [35,38,42,44,45,48,51] that used online evaluation, 6 studies [35,39,40,42,43,49] evaluated the diet-related HRSs with target users of patients, while 3 studies evaluated the HRSs that relied on experts, such as nutritionists [40,46] and dietitians [49]; their main roles were to validate the recommendation results. Four of the 5 offline evaluation studies [39,40,43,46,47] used the same dataset for training and evaluation [41,44,47,48].

## Discussion

### Principal Findings

This scoping review revealed that diet-related HRSs for individuals with chronic conditions were still in their early stages, with limited patient-specific designs and significant room for improvement. The review highlighted substantial gaps in target users, system functions, recommendation content, recommendation feature implementation, and evaluation approaches, which need to be addressed to support the development of more effective and patient-centered diet-related HRSs.

### Comparison with Prior Work

#### Target Users of Diet-Related HRSs

The target users of diet-related HRSs were mainly patients with single or complex chronic health conditions. They not only need dietary recommendations tailored to their preferences [39,41,42,44,48,50], but most importantly, their health conditions [38,40,45,46,51], highlighting the need for more disease-specific solutions. Age groups are another factor that needs to be considered, for example, older adults often face challenges such as limited mobility, cognitive decline, and changes in appetite or food preferences [40,42], while sick children and their caregivers face unique dietary needs due to treatment-related restrictions and appetite loss [52,53]. Therefore, balancing disease-related requirements with personal needs is fundamental to developing high-quality diet-related HRS. It is also essential to support caregivers in preparing meals that are nutritionally appropriate and medically compliant.

#### Function Structures of Diet-Related HRSs

The user information component enables diet-related HRSs to collect basic demographic and health data, which supports the generation of tailored dietary recommendations [35,38-40]. By incorporating dynamic inputs such as food tracking and activity logs, the system can further provide personalized, context-sensitive advice with timely feedback and adjustments based on patients’ health progress [43,54]. Knowledge and decision support functions served as a critical layer in enhancing the intelligence and reliability. For example, by integrating chronic kidney disease calculators [35], nutrient search engines [45], and expert knowledge bases [39], these systems move beyond simple food suggestions to provide evidence-based recommendations. This clarified how the nutritional rationale, which potentially enhances patients’ understanding and trust, long-term adherence, and sustainable dietary behavior change [44,45]. The accuracy and relevance of food or diet recommendations depend on the robustness of the algorithm used to process the data and the diversity of food or recipes options integrated into the system [55-57]. Data management and additional interactive functions have enhanced the usability and clinical relevance of diet-related HRSs and supported user engagement, empowering patients to actively and continuously manage their dietary self-care.

#### Recommendation Content of Diet-Related HRSs

Patients’ needs for the content of diet-related HRSs are multidimensional. Beyond simple food lists, an effective diet-related HRS must provide actionable guidance on cooking methods, ingredient combinations, and personalized meal plans [39,42-44,47,48,50]. Well-structured recipes emerge as a key feature, as they help users visualize meal preparation and improve understanding of the nutritional rationale behind recommendations [44]. Such transparency fosters engagement and adherence, particularly when supported by nutrition professionals [58,59]. Additionally, offering varied recipe options allows patients to personalize their meal plans according to their tastes, dietary restrictions, and the availability of ingredients [39]. This flexibility caters to users with complex health conditions and empowers them with a sense of control, which is vital for sustaining long-term dietary management [42].

#### Implementation of Recommendation Features in Diet-Related HRSs

##### Recommendation Methods

In diet-related HRSs, hybrid approaches appear particularly valuable for balancing clinical appropriateness with individual preferences to promote long-term adherence. In addition, adaptability is an essential feature, allowing systems to respond to changing health conditions, behaviors, and user needs. As patients’ health status and preferences evolve, systems such as “SHARE” demonstrate the value of dynamic updates that allow users to refine their meal plans, making the system more user-centered and responsive [42]. However, a critical gap across the reviewed studies is the insufficient consideration of medication-food interactions. For patients with chronic conditions, clinically significant interactions such as warfarin-vitamin K [60], angiotensin-converting–enzyme inhibitors-potassium [61], or metformin-alcohol [62], carry substantial risks. Future diet-related HRSs should adopt hybrid approaches that integrate drug-nutrient knowledge and dynamic monitoring with preference learning, ensuring recommendations that are both personalized and clinically safe.

##### Recommendation Technology

Appropriate recommendation technology can transform users’ vague needs into clear ones and filter out irrelevant information [63]. HyR that combines CF, CB, and KB methods is particularly effective. These methods overcome traditional limitations such as cold start and data sparsity by integrating multiple information sources, including nutritional knowledge and disease-specific data [64]. Notably, KB and KG-driven algorithms were especially well-suited for diet-related HRSs. By incorporating expert knowledge and medical guidelines, they ensure scientific accuracy, clinical reliability, and alignment with patients’ dietary management goals, resulting in more comprehensive and robust systems [65].

##### Data Sources

The effectiveness of diet-related HRSs largely depends on the quality and appropriateness of data sources. Patient data mainly come from personal input, including medical records, treatment history, and sociodemographic information [35,38-45,47-49]. Incorporating dietary behavior data (eg, meal logs, food purchase records, and subjective reports) and clinical data (eg, diagnosis, treatment, and disease stage) helps ensure that dietary recommendations are both personalized and medically appropriate [66,67]. Although patient-specific data are essential for personalized recommendations [35,44], sensitive medical and behavioral data entail serious privacy, security, and ethical risks. Given patients’ fragile health status and dietary restrictions, dietary data from professional resources, such as the Nutrition Division of the Ministry of Public Health (MOPH) [46], the official website of the composition of foods integrated dataset (CoFID) [48], and the Japan Preventive Association of Lifestyle-related Disease (JPALD) [45], are vital for ensuring the accuracy of recommendations. In contrast, nonprofessional sources such as general cooking websites are not recommended in clinical contexts due to their limited reliability, particularly for patients with complex conditions.

##### Implementation Process

The Association for Computing Machinery has issued guidelines for designing and evaluating RSs [68]. However, no widely accepted standards have been established for implementing HRSs, especially diet-related HRSs. This gap highlights challenges arising from disease diversity, complex food preferences, and context-dependent factors that are difficult to acquire or simulate [28]. The reviewed systems generally followed a similar workflow, involving user profiling, structured knowledge integration, personalized filtering, and ranking, reflecting a growing understanding of how to align dietary recommendations with individual health needs and preferences. The integration of advanced recommendation technologies, such as ontology-based reasoning [51], optimization algorithms (eg, ant colony methods [48]), Long Short-Term Memory–based dynamic modeling [44], and KGs [39], has advanced more intelligent and context-aware systems. However, the adoption of these technologies remains limited, and few studies have systematically evaluated their impact on recommendation quality or patient outcomes.

### Evaluation of Diet-Related HRSs

#### Evaluation Criteria

In diet-related HRSs, accuracy has been the primary evaluation criterion, typically evaluated by prediction score accuracy, prediction score correlation, classification accuracy, and sorting accuracy [69]. However, the rationality of the recommendations has received limited attention. None of the 15 reviewed studies [35,38-51] evaluated the scientific soundness of food or recipe recommendations. While health care providers were commonly involved in evaluating the recommendation results, official institutional input on content was rare, revealing a major gap in ensuring the credibility and reliability of recommendations.

Beyond accuracy and content rationality, the effectiveness of diet-related HRSs is closely tied to their ability to promote healthy eating behaviors. The key goal of the diet-related HRSs is to empower users to make informed dietary choices and motivate healthier behaviors [70]. Existing evaluations of behavior change have primarily examined improvements in diet quality and diversity, measured by the China Elderly Dietary Guidelines Index and the dietary diversity score [50]. Although user engagement and personalized feedback were recognized as essential for sustaining behavior change, few studies evaluated long-term effects on patients’ dietary behaviors.

Moreover, clinical trials in real-world health care settings remain lacking; even the study [39] involving patients was an observational cohort study rather than a clinical trial, limiting statistical power and generalizability. Additionally, no studies examined the cost-effectiveness of implementing diet-related HRSs in health care settings, which is crucial for adoption in resource-constrained healthcare systems.

#### Evaluation Methods and Process

The evaluation of diet-related HRSs can be carried out using various methods, each with its advantages and limitations. Online evaluations, which assess system performance through real-time user feedback or surveys, provide valuable insights into user interactions but often involve higher costs [22]. Offline evaluations in controlled settings are more feasible but have limited external validity due to their inability to reflect real-world usage [22,71]. Emerging approaches such as online A or B testing enable rapid, cost-effective comparisons of different system versions, helping predict broader performance even with small samples [72,73].

Diet-related HRS evaluations generally follow a staged approach from development to postdeployment. During development, stakeholder involvement in usability evaluation is critical to ensure user-centered design [74]. After development, accurate assessments verify that recommendations are reliable and relevant to target users [35,41,42,44,46-49,51]. Postdevelopment, gathering user feedback on effectiveness and satisfaction is essential for refining the system and improving its responsiveness to user needs [35,39,40,42,43,46]. These stages form a comprehensive evaluation process ensuring the reliability and usability of diet-related HRSs.

Although several reviews have examined HRSs, few have focused specifically on diet-related HRSs. One scoping review of 36 studies identified only 8 diet-focused HRSs [75]. A systematic review of 73 studies identified 26 nutrition-related systems, most of which targeted populations without strict medical dietary requirements, thereby revealing the lack of disease-specific syntheses [22]. Existing diet-related HRS reviews have primarily addressed technical aspects, such as semantic interoperability [76], explainable artificial intelligence [77], and data-driven personalization methods [78]. They offer useful technical insights but rarely address disease-specific dietary needs or the related clinical and safety challenges. In contrast, our review focuses on diet-related HRSs for patients with chronic conditions. It highlights key requirements such as clinical data integration, dynamic health monitoring, tailored recipe-level recommendations, and persistent gaps in evaluating behavior change and clinical outcomes. These disease-focused insights extend prior work and inform the development of more personalized and clinically reliable diet-related HRSs.

### Future Directions

Future research should prioritize developing diet-related HRSs based on a user-centered design framework. These systems must be tailored to patients’ specific needs with a deep understanding of their cognitive abilities, digital literacy, cultural dietary preferences, and daily routines. Diet-related HRSs need to address clinical challenges through contextually relevant functional designs, customized for different patient groups, from older adults with multimorbidity to pediatric patients requiring caregiver-mediated support. Practical usability features, such as adaptive reminders, contextualized recipe guidance, and dynamic feedback loops, are essential for real-world effectiveness.

To support patients’ sustained dietary behavior change, recommendations should move beyond static food lists toward evidence-based, actionable guidance using practical tools such as portion guides, ingredient lists, and meal preparation videos [79]. Future systems should adopt hybrid, adaptive recommendation mechanisms and integrate with electronic health records (EHRs) and mobile apps to enhance personalization, interpretability, clinical relevance, and engagement, which is essential for sustaining long-term adherence to dietary changes. Additionally, incorporating behavior change theories, such as Social Cognitive Theory [80], Theory of Planned Behavior [81], and the Fogg Behavior Model [82], can strengthen the dietary intervention design. Gamification elements informed by these theories, such as rewards and rankings, may further motivate users and increase adherence [83].

When managing sensitive health data, especially when integrating with EHRs and personal medical records, the systems must implement robust data governance, including secure storage, encryption, role-based access, and transparent consent. Compliance with regulations such as the US Health Insurance Portability and Accountability Act (HIPAA) [84], the European Union’s General Data Protection Regulation (GDPR) [85], and national standards are essential to safeguard patient privacy and foster user trust. Finally, standardized evaluation methods and robust frameworks, combining clinical trials, behavior change evaluation, and usability evaluation, are essential to verify effectiveness, accuracy, and long-term impact in real-world settings, while ensuring cost-effectiveness and compliance (eg, US Food and Drug Administration approval and European Conformity marking) for safe and scalable healthcare implementation.

### Implication for Clinical Practice

For clinicians and dietitians, diet-related HRSs can serve as supportive tools to deliver personalized, evidence-based, and adaptive dietary guidance aligned with patients’ medical conditions, treatment regimens, cultural preferences, and daily routines. By incorporating medication-food interaction checks and actionable educational content, these systems can enhance patient safety, dietary adherence, and long-term health management. Future diet-related HRSs could integrate seamlessly into clinical workflows and demonstrate measurable health outcomes. Embedding diet-related HRSs within EHRs would ensure that dietary recommendations are consistent with medications, lab results, and care plans. By linking dietary adherence to physiological indicators such as hemoglobin A_1c_, blood pressure, or lipid levels, systems can enable real-time feedback and timely clinical intervention. Beyond accuracy, future evaluations should predefine clinical end points (eg, hemoglobin A_1c_ reduction or blood pressure control) and validate effectiveness through randomized controlled trials and real-world studies.

### Limitations

This study has several limitations. First, only studies published from January 2010 to October 2024 were included, which may not reflect recent developments. Second, only English and Chinese literature were reviewed, excluding studies in other languages. Third, the focus was primarily on chronic health conditions, with limited exploration of diet-related HRSs for acute conditions or general wellness, suggesting a need for broader research in future studies. In addition, the included studies themselves demonstrated certain methodological limitations, such as small sample sizes, reliance on offline testing, and limited evaluation of actual behavior change. These issues reflect not only the limitations of this review but also highlight gaps in the existing research that warrant more rigorous clinical evaluation.

### Conclusions

Diet-related HRSs can offer personalized dietary recommendations for patients with chronic conditions. The analysis reveals significant gaps, demanding that future systems be grounded in user-centered design to meet patient-specific needs. These systems must recommend more practical and actionable dietary guidance. The adoption of hybrid recommendation techniques can enhance the precision and clinical relevance of dietary recommendations. Establishing standardized evaluation metrics and conducting real-world studies with long-term follow-up will be essential. This will verify the system’s ability to positively change dietary behaviors and improve clinical outcomes. Addressing these issues will transform diet-related HRSs into trustworthy and impactful tools for managing chronic diseases and delivering patient-centered care.

## Supplementary material

10.2196/77726 Multimedia Appendix 1 Search strategy.

10.2196/77726 Multimedia Appendix 2 The publication years and countries of the included studies.[Author-notes equal-contrib1]

10.2196/77726 Checklist 1 PRISMA-ScR Checklist.

## Abbreviations

CB: content-based
CF: collaborative filtering
CoFID: composition of foods integrated dataset
EHR: electronic health record
GDPR: General Data Protection Regulation
HIPAA: Health Insurance Portability and Accountability Act
HRS: Health Recommender System
HyR: hybrid recommendation
JPALD: Japan Preventive Association of Lifestyle-related Disease
KB: knowledge-based
KG: knowledge graph
MOPH: Ministry of Public Health
PRISMA: Preferred Reporting Items for Systematic Reviews and Meta-Analyses
PRISMA-ScR: Preferred Reporting Items for Systematic Reviews and Meta-Analyses Extension for Scoping Reviews
RS: Recommender System
