# Supplementary material for: Diet-Related Health Recommender Systems for Patients With Chronic Health Conditions: Scoping Review
Source: J Med Internet Res. 2026 Jan 14;28:e77726. doi: 10.2196/77726 (PMC12809011; doi:10.2196/77726)
Supplement: Multimedia Appendix 1 [file jmir-v28-e77726-s001.pdf]

NO.	Detail	Results
	PubMed	
1	Search: "Recommender system*"[title/abstract] OR "Hybrid recommendation*"[title/abstract] OR "Collaborative filtering"[title/abstract] OR "Content based recommendation*"[title/abstract] OR "Recommendation* system*"[title/abstract] OR "Knowledge based recommendation*"[title/abstract]	1797
2	Search: "Recipe*"[Title/Abstract] OR "Diet*"[Title/Abstract] OR "Food"[Title/Abstract] OR "Eat* "[Title/Abstract] OR " Nutrition*"[Title/Abstract]	1,594,783
3	(#1 AND #2) AND (("2010/01/01"[Date - Publication] : "2024/10/31"[Date - Publication]))	81

Search	Actions	Details	Query	Results	Time
#3	•••	>	Search: **(#1 AND #2) AND (("2010/01/01"[Date - Publication] : "2024/10/31"[Date - Publication]))**	81	16:04:28
#2	•••	>	Search: **"Recipe*"[Title/Abstract] OR "Diet*"[Title/Abstract] OR "Food"[Title/Abstract] OR "Eat "[Title/Abstract] OR " Nutrition*" [Title/Abstract]**	1,594,143	16:03:13
#1	•••	>	Search: **"Recommender system*"[title/abstract] OR "Hybrid recommendation*"[title/abstract] OR "Collaborative filtering" [title/abstract] OR "Content based recommendation*" [title/abstract] OR "Recommendation* system*"[title/abstract] OR "Knowledge based recommendation*"[title/abstract]**	1,797	16:02:08

NO.	Detail	Results
	Medline	
1	(Recommender system* or Hybrid recommendation* or Collaborative filtering or Content based recommendation* or Recommendation* system*).ab.	888
2	limit 1 to yr="2010 - 2024"	730
3	(Recipe* or Diet* or Food or Eat* or Nutrition*).ab.	1211703
4	limit 3 to yr="2010 - 2024"	714708
5	1 and 2 and 3 and 4	55

Search History (5) View Saved

#	Searches	Results	Runtime	Type	Actions		Annotations
1	(Recommender system* or Hybrid recommendation* or Collaborative filtering or Content based recommendation* or Recommendation* system*).ab.	888	1.10	Advanced	Display Results	More	
2	limit 1 to yr="2010 - 2024"	730	0.19	Advanced	Display Results	More	
3	(Recipe* or Diet* or Food or Eat* or Nutrition*).ab.	1211703	0.49	Advanced	Display Results	More	
4	limit 3 to yr="2010 - 2024"	714708	0.67	Advanced	Display Results	More	
5	1 and 2 and 3 and 4	55	0.09	Advanced	Display Results	More	

NO.	Detail	Results
	Embase	
1	(Recommender system* or Hybrid recommendation* or Collaborative filtering or Content based recommendation* or Recommendation* system* or Knowledge based recommendation*).ab.	1819
2	(Recipe* or Diet* or Food or Eat* or Nutrition*).ab.	1899488
3	1 And 2	119
4	limit 3 to yr="2010 - 2024"	96

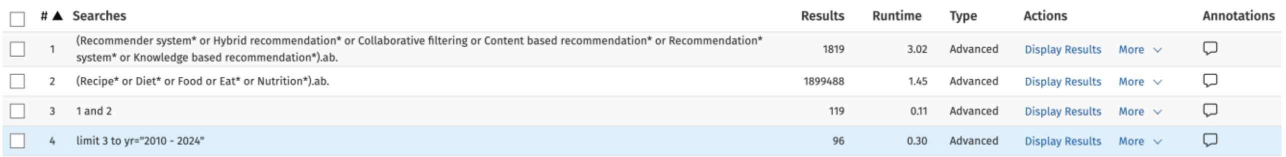

	Web of Science	
1	All Fields =(Recommender system* or Hybrid recommendation* or Collaborative filtering or Content based recommendation* or Recommendation* system* or Knowledge based recommendation*)	4,205,423
2	Title =(Recipe* or Diet* or Food or Eat* or Nutrition*)	981,061
3	#1 AND #2 AND 2010-01-01/2024-10-31 (Publication Date)	696

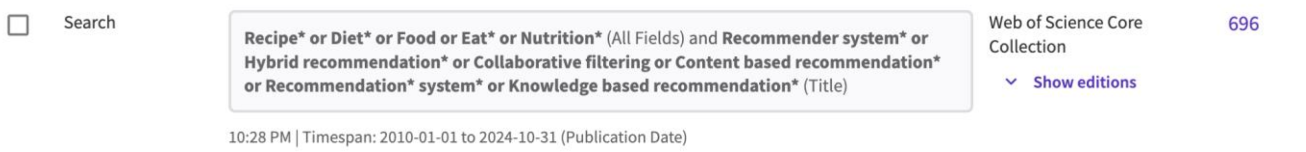

	IEEE Xplore	
1	("Abstract":Recommender system*) OR ("Abstract":Hybrid recommendation*) OR ("Abstract":Collaborative filtering) OR ("Abstract":Content based recommendation*) OR ("Abstract":Recommendation* system*) OR ("Abstract":Knowledge based recommendation*)	35,188
2	("Abstract":Recipe*) OR ("Abstract":Diet*) OR ("Abstract":Food) OR ("Abstract":Eat*) OR ("Abstract":Nutrition*)	28,142
3	1 AND 2 AND 2010-2024	870

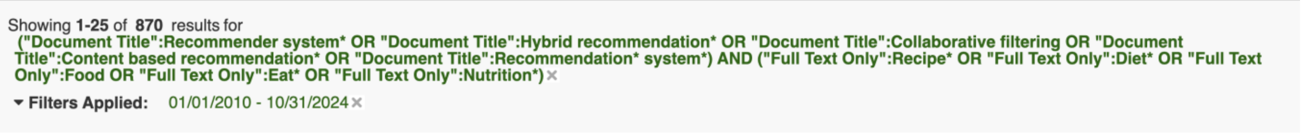

	CINAHL	
1	XB (Recommender system*) OR XB (Hybrid recommendation*) OR XB (Collaborative filtering) OR XB (Content based recommendation*) OR XB (Recommendation* system*) AND XB (Knowledge based recommendation*)	311
2	XB (Recipe*) OR XB (Diet*) OR XB (Food) OR XB (Eat*) OR XB (Nutrition*)	378,924
3	1 AND 2 AND 2010/01/01-2024/10/31	24

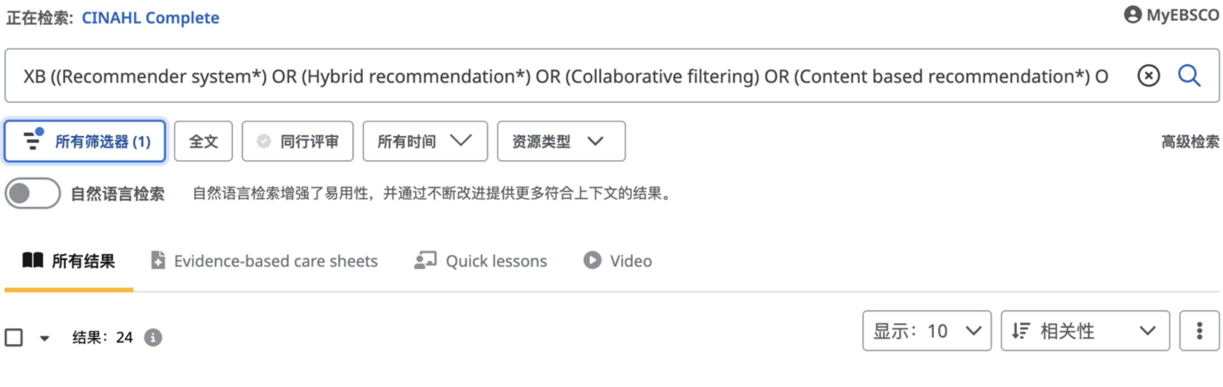

	SinoMed	
1	("推荐系统"[摘要:智能] OR "个性化推荐"[摘要:智能] OR "推荐算法"[摘要:智能] OR "推	2192

	荐引擎"[摘要:智能] OR "算法模型"[摘要:智能] OR "健康管理系统"[摘要:智能] OR "智能化管理"[摘要:智能])	
2	("食谱"[摘要:智能] OR "菜谱"[摘要:智能] OR "餐谱"[摘要:智能] OR "食物"[摘要:智能] OR "饮食"[摘要:智能] OR "营养"[摘要:智能] OR "配餐"[摘要:智能])	448312
3	1 AND 2 AND 2010-2024[日期]	36

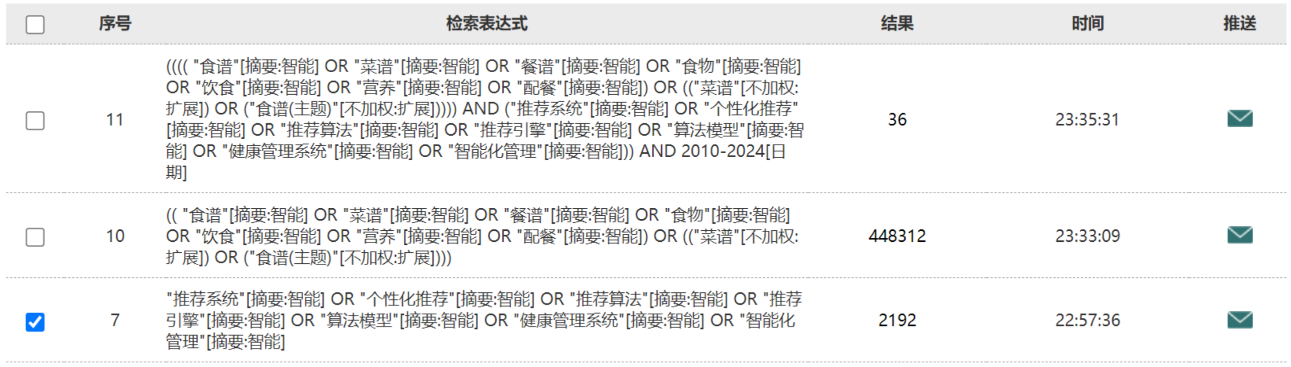

	CNKI	
1	AB = '推荐系统' + '个性化推荐' + '推荐算法' + '推荐引擎' + '算法模型' + '健康管理系统' + '智能化管理'	75824
2	AB = '食谱' + '菜谱' + '餐谱' + '食物' + '饮食' + '营养' + '配餐'	1291857
3	AB = ('食谱' + '菜谱' + '餐谱' + '食物' + '饮食' + '营养' + '配餐') AND AB = ('推荐系统' + '个性化推荐' + '推荐算法' + '推荐引擎' + '算法模型' + '健康管理系统' + '智能化管理')	710

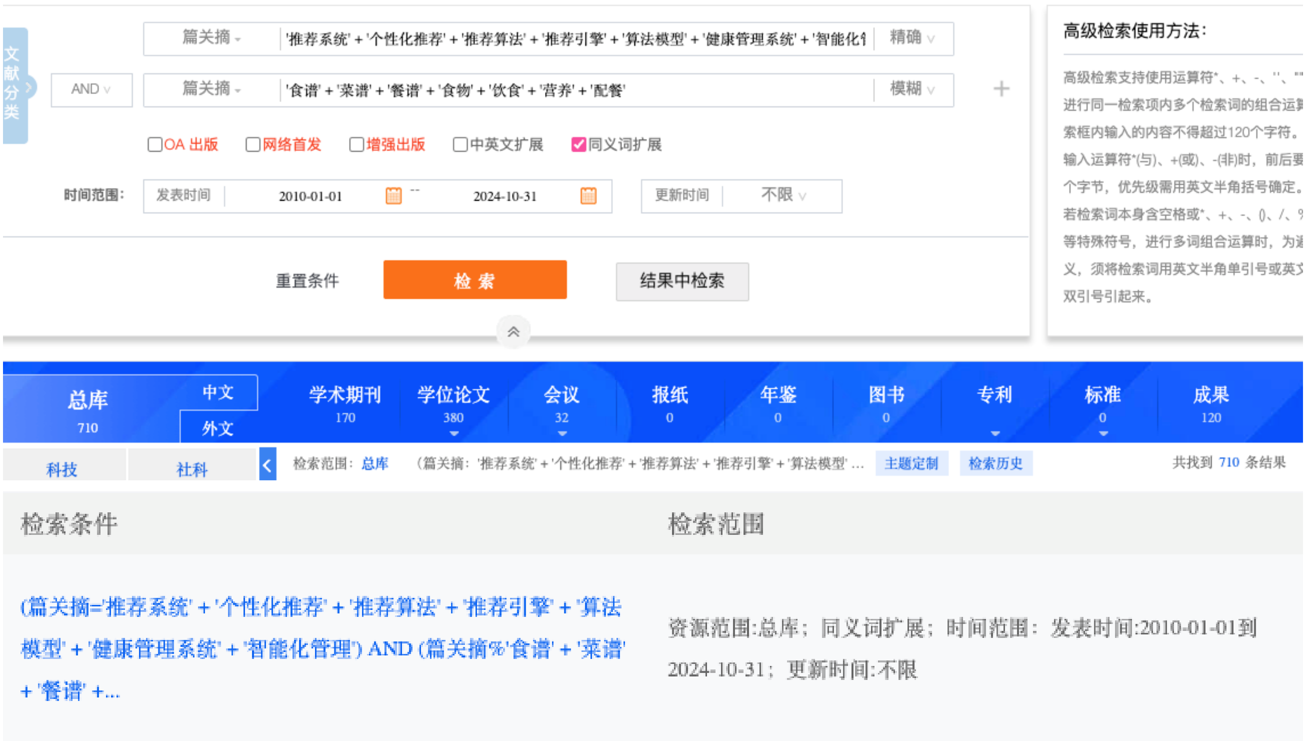

	Wanfang	
1	(题名或关键词:(推荐系统 OR 个性化推荐 OR 推荐算法 OR 推荐引擎 OR 算法模型 OR 健康管理系统 OR 智能化管理)) and 发表时间:*-2024	362199
2	(摘要:(食谱 OR 菜谱 OR 餐谱 OR 食物 OR 饮食 OR 营养 OR 配餐)) and 发表时间:*-2024	1049393
3	1 AND 2 AND 2010-2024	1211

检索式	检索结果
(摘要:(食谱 OR 菜谱 OR 餐谱 OR 食物 OR 饮食 OR 营养 OR 配餐) and 题名或关键词:(推荐系统 OR 个性化推荐 OR 推荐算法 OR 推荐引擎 OR 算法模型 OR 健康管理系统 OR 智能化管理)) and 发表时间:2010-2024	1212
(摘要:(食谱 OR 菜谱 OR 餐谱 OR 食物 OR 饮食 OR 营养 OR 配餐)) and 发表时间:*-2024	1049393
(题名或关键词:(推荐系统 OR 个性化推荐 OR 推荐算法 OR 推荐引擎 OR 算法模型 OR 健康管理系统 OR 智能化管理)) and 发表时间:*-2024	362199

	VIP Chinese Journal database	
1	主题=食谱 OR 菜谱 OR 餐谱 OR 食物 OR 饮食 OR 营养 OR 配餐	1,029,219
2	摘要=推荐系统 OR 个性化推荐 OR 推荐算法 OR 推荐引擎 OR 算法模型 OR 健康管理系统 OR 智能化管理	558,516
3	1 AND 2 AND 2010-01-2024-10	703

序号	检索式	附加条件	检索结果
1	篇名=食谱 OR 菜谱 OR 餐谱 OR 食物 OR 饮食 OR 营养 OR 配餐 AND 摘要=推荐系统 OR 个性化推荐 OR 推荐算法 OR 推荐引擎 OR 算法模型 OR 健康管理系统 OR 智能化管理	文献类型：期刊<br>时间范围：2010-01至2024-10<br>期刊来源：	703
