# Supplementary figures and images for: Diet-Related Health Recommender Systems for Patients With Chronic Health Conditions: Scoping Review
Source: J Med Internet Res. 2026 Jan 14;28:e77726. doi: 10.2196/77726 (PMC12809011; doi:10.2196/77726)

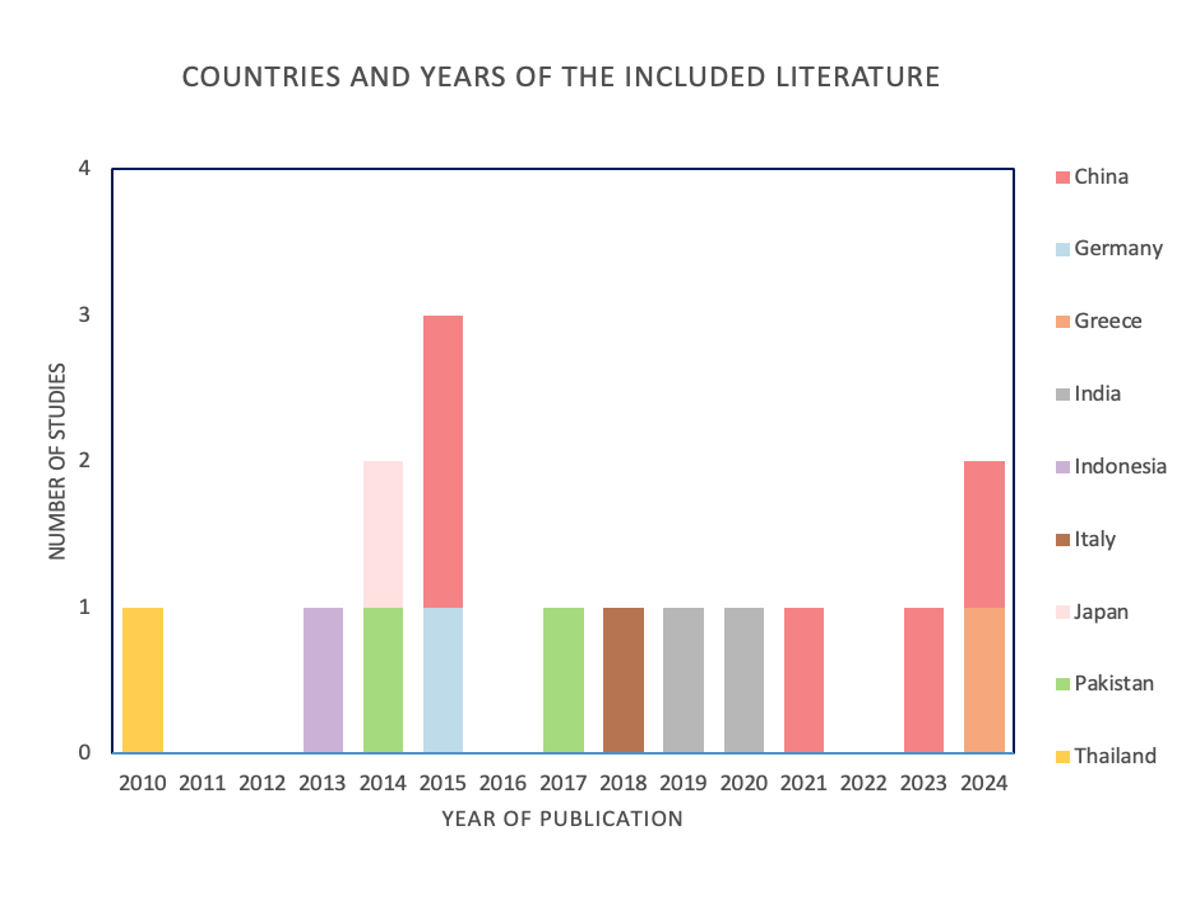

Supplement: Multimedia Appendix 2 [file jmir-v28-e77726-s002.png]
